# SNP algorithms for prediction of efficacy and adverse events of abatacept (ABT)

**DOI:** 10.1186/ar3649

**Published:** 2012-02-09

**Authors:** James E Middleton, Tsukasa Matsubara, Keiko Funahashi, Satoru Koyano, Takafumi Hagiwara, Takako Miura, Kosuke Okuda, Takeshi Nakamura, Mitsuyoshi Iwahashi, Tomomi Tsuru, Shoichi Uchimura, Shigeru Honjo

**Affiliations:** 1 Research Institute of Joint Diseases, Kobe, Japan; 2 Matsubara Mayflower Hospital, Kato, Japan; 3 Higashi-Hiroshima Memorial Hospital, Higashi-Hiroshima, Japan; 4 PS Clinic, Fukuoka, Japan; 5 Kanzaki Municipal General Hospital, Japan; 6 Honjo Rheumatism Clinic, Japan

## Background

Abatacept (ABT), a CTLA4-Ig fusion protein, which inhibits the binding of CD28 and CD80 agents targeted to T-cells, is a relatively new biological agent for RA treatment in Japan. However, there is no method for prediction of responders, non-responders, or adverse events which can occur during treatment. We established SNP algorithms for prediction of responders (R) or non-responders (NR), and adverse events in ABT-treated patients.

## Materials and methods

Forty-six RA patients treated with ABT were included in this study. Efficacy was assessed by DAS28 (CRP) at 48 weeks after the initial treatment. Any adverse events that may have been related to ABT administration and observed at 48 weeks of this long-term administration and during phase II were considered to be side effects. Genome-wide SNP genotyping was performed by Illumina Human610-Quad chip technology. Case-control analyses between 598,821 SNPs and responsiveness or occurrence of adverse events were examined by Fisher's exact test. We selected 10 SNPs associated with ABT-responsiveness, remission, and adverse events (p < 0.0001). We scored the relationship between each SNP and responsiveness, the estimated total score of 10 SNPs (estimated scoring in each SNP was as follows: homo allele in the majority in responders: +1 point, hetero allele: 0 points, and homo allele in the majority of non-responders: -1 point), and then examined relationships between responders and non-responders, remission and non-remission, and occurrence of adverse events, plus or minus, and the total score.

## Results

Accuracy, specificity, and sensitivity of the algorithm for responsiveness of abatacept ranged from 90-96%. For remission, accuracy, specificity and sensitivity of the algorithm ranged from 91-97%. For adverse events, accuracy, specificity and sensitivity of the algorithm ranged from 95-100%. It is therefore suggested that the SNP algorithms can predict responders and adverse events prior to the initiation of treatment with abatacept.

## Conclusions

These highly accurate algorithms using SNP analysis may be useful in the prediction of responsiveness and adverse events before treatment with abatacept, and in this way can contribute to future tailor-made treatment with biologic agents.

